# Variations in Parent and Teacher Ratings of Internalizing, Externalizing, Adaptive Skills, and Behavioral Symptoms in Children with Selective Mutism

**DOI:** 10.3390/ijerph16214070

**Published:** 2019-10-23

**Authors:** Evelyn R. Klein, Cesar E. Ruiz, Kylee Morales, Paige Stanley

**Affiliations:** Department of Communication Sciences and Disorders, La Salle University, Philadelphia, PA 19141, USA; moralesk5@student.lasalle.edu (K.M.); stanleyp3@student.lasalle.edu (P.S.)

**Keywords:** selective mutism, behavioral rating scales, multiple informants, assessment, shyness

## Abstract

Selective mutism (SM) is an anxiety disorder that impacts communication. Children with SM present concerns to parents and teachers as they consistently do not speak in situations where there is an expectation to speak, such as at school, but speak in other settings where they feel more comfortable, such as at home. The purpose of this study was to investigate the relationship between parents’ and teachers’ perceptions of children with SM on behavioral rating scales and language measures. Forty-two children (22 boys and 20 girls, ranging from 2.4 to 13.8 years, with a mean age of 7.1 years) took part in this study. Parents and teachers completed the Behavior Assessment System for Children (BASC-3) measuring internalizing behaviors, externalizing behaviors, adaptive skills, and behavioral symptoms. Frequency of speaking and language abilities were also measured. Parents and teachers both identified withdrawal as the most prominent feature of SM but parents saw children as significantly more withdrawn than did their teachers. Both rated children similarly at-risk on scales of functional communication and social skills. Higher adaptive skills (including functional communication and social skills) were positively correlated with vocabulary, narrative language, and auditory serial memory according to teachers. Parent and teacher rating scales provide valuable information for diagnosis and progress monitoring. Children with SM can benefit from mental health practitioners who can identify and enhance their emotional well-being.

## 1. Introduction

### 1.1. Selective Mutism and Comorbidities

Selective Mutism (SM) is identified as an anxiety disorder in the Diagnostic and Statistical Manual of Mental Disorders, Fifth Edition (DSM-5) [1]. Individuals with SM display a consistent failure to speak in specific situations where there is an expectation to speak, such as at school. Individuals speak in other situations where they feel more comfortable, such as at home. SM interferes with social communication and educational achievement. For the diagnosis, symptoms must persist beyond the first month of school and the failure to speak is not due to lack of knowledge or comfort with spoken language expected within the social situation. Additionally, the disturbance is not better identified by a communication disorder and it does not occur exclusively with autism spectrum disorder (ASD), psychosis, or schizophrenia. The prevalence varies but is generally between 0.2% and 0.8% [2], less than one percent of children in the United States. More recently, SM is approaching equivalence in boys and girls but has been slightly more common in girls than boys [3].

Comorbidities have been identified such as speech and language problems, obsessive compulsive disorder, attention deficits, depression, panic, and social phobia, to name several. In a retrospective chart review study by Steffenburg, Steffenburg, Gillberg, and Billstedt [4], which included 97 individuals diagnosed with SM, 61 of them (63%) met criteria for ASD. Of this group, 4% had what would be referred to as the Asperger’s type. Medical records indicated that 27% had delayed or abnormal development for speech and language. Ninety-five percent of the children did not display SM symptoms at home.

As treatment professionals working with children who do not readily speak, a clinician should obtain information from parents and teachers. Once a child is referred, clinicians rely not only on their own observations, but reports from parents and other professionals in the child’s life to help accurately diagnose the disorder. Then they can develop appropriate plans for treatment. For many disorders, especially SM, an early, accurate diagnosis is crucial for treatment planning. Concomitant disorders can certainly impact the trajectory of SM treatment.

### 1.2. Anxiety and Disorders Related to Selective Mutism

Withdrawal and avoidance are common symptoms of SM. Children and adolescents with SM describe fears that fall into four main categories: (1) consistent focus on the physical symptoms of their anxiety, (2) consistent focus on their thoughts, (3) overwhelming feelings of anxiety, and (4) avoidance mechanisms (mutism) to help alleviate their symptoms [5]. Some individuals with SM remain completely silent in uncomfortable settings while others speak in a different manner using a unique voice, whispering, or speaking as a much younger person. The onset of SM can be gradual but usually occurs before the age of five years old [6].

In a study by Capozzi, Manti, Dio Trani, Romani, Vigliante, and Sotos [7], 58 preschool children with SM or generalized anxiety disorder (GAD) and their parents were evaluated at a pediatric clinic over a period of five years. Child and family variables did not discriminate children in the SM and GAD groups. Using clinical interviews and self-report questionnaires such as the Child Behavior Checklist (CBCL), children with SM scored higher on withdrawal and lower on aggressiveness than children with GAD. Using a parental psychological profile (SCL-90-R), parents of children with SM scored significantly higher on stressful life events than parents of children with GAD. The SM group was more frequently described as isolated and avoidant. 

Social phobia has also been related to social anxiety [8] and is defined in the DSM-5 as a persistent fear of embarrassment or humiliation in one or more social or performance situations, especially with peers. Exposure to the feared social situation almost invariably provokes anxiety, panic, and may be expressed by crying, tantrums, freezing, or avoiding social situations with unfamiliar people. Like SM, its onset is gradual [6].

Social anxiety is frequently viewed on a spectrum ranging from mild anxiety to an actual social anxiety disorder that affects the individual’s ability to function [9]. One article suggests that shyness is a mild point on that spectrum, defining it as a form of excessive self-focus with preoccupation of thoughts, feelings, along with physical reactions that can vary from mild social awkwardness to social phobia [10]. Because SM is listed as an anxiety disorder in the DSM-5 with symptoms severely affecting the child’s ability to function in social situations, some people view SM as being at the extreme end of the social anxiety spectrum [11]. 

### 1.3. Differentiating Shyness from Selective Mutism

Shyness and SM are often confused. While SM, social anxiety, and social phobia have clinical definitions and suggestions for treatment, shyness does not. Shyness is not a clinically defined disorder. Rather it is characterized as a personality trait which hinders children’s ability to interact and communicate with others. Shyness may still evoke somatic symptoms (such as trembling, sweating, blushing), cognitive symptoms (for example, fear of negative evaluation), and behavioral symptoms (avoidance of social situations). Nevertheless, shyness is viewed as more common and less problematic than SM [8]. A fundamental difference between a shy child and a child with SM is that a shy child is able to speak, even minimally, in public contexts when needed, whereas a child with SM is often unable to do so [12]. 

People who are shy have been identified as reserved, timid, and quiet. According to some, being shy is a social phenomenon that is experienced during interactions [13]. Shyness is thought to be related to one’s fears of negative evaluation from others that leads to avoidance. Compared to socially anxious individuals, shy people tend to think they do poorly in their attempt to meet the standards and expectations of effective social behavior [14]. There is usually an added concern about being rejected. Anxiety is more likely to arise when a person’s perception of the social self does not match the perceived social evaluations from others. Shyness and anxiety are not synonymous [15]. However, both tend to impact internalizing behavior problems. 

Children who are shy tend to produce shorter utterances during informal conversations when compared to their age-normed peers. However, their language skills remain within average range [16,17]. This is not the case for children with SM. In many children with SM, a contributing factor to their silence may be an inability to effectively use language to express themselves in their environments. This factor is compounded by children’s anxiety and makes vocalization difficult. That is the ability to produce voice by adducting the vocal cords [18]. Children with SM have been found to demonstrate shorter and less detailed utterances than same-aged peers without SM when producing a narrative story. Their utterances were noticeably less complex and included fewer clauses [19]. Children with SM demonstrated expressive language deficits during standardized language assessment [20].

Shyness and SM are not equivalent. For those with SM, there is failure to speak in specific social situations but normal speech in other settings, such as at home with immediate family. Shy children are more likely to speak even minimally [21]. Chronic shyness appears to be related to negative outcomes such as low self-esteem, loneliness, and negative affect [10]. It is important to identify SM early in a child’s life so that appropriate treatment can diminish negative long-term effects.

### 1.4. Parents and Teachers as Informants for Selective Mutism

Parents and teachers are essential to the assessment and treatment of children with SM. Once a child is referred for an evaluation, clinicians continue to rely on parents and teachers to provide insight about the child. Scales and questionnaires are an informative and useful source to further understand children outside the clinical setting. To reliably and validly use questionnaires, one must know the accuracy of parents’ and teachers’ portrayals of a child. Current information relates parents’ and teachers’ ratings of children with a variety of diagnoses but little research has explored parent–teacher congruence when rating children with SM.

Informants with the same role (e.g., parent and parent, teacher and teacher) have significantly higher congruence than those with different roles (e.g., teacher and parent) [22]. This is likely due to the fact that similar informants observe children in similar settings and have similar relationships to the child. However, even similar raters report behaviors in different ways. A study by Möricke, Buitelaar, and Rommelse [23] found that when completing the Social Communication Questionnaire, both parents of the preschool participants had fair interrater reliability, but that mothers were more likely to report social communication problems in their children than fathers. 

Teachers’ perspectives are also of great value to clinicians when gathering information about children’s behavior for diagnostic purposes. Teacher perceptions are necessary to achieve a holistic understanding of a child. They provide a view of the child in a setting that is unfamiliar and more structured [24]. In the school setting, children have increased opportunities to socialize and use language in a more functional way with more peers than adults. Furthermore, teachers have been shown to have accurate perspectives on student academic achievement which may generalize to their accuracy about children’s behaviors. A meta-analysis involving 75 studies revealed a high positive correlation between teachers’ judgments and students’ actual academic performance [25]. 

### 1.5. Discrepancies between Parents and Teachers

There are important implications for discrepancies between multiple informants. Discrepancies can highlight problems in identifying, diagnosing, and treating children [4,26]. Two contradicting presentations of the child [26] can create challenges but they can add to a treatment plan for a child who has been reported to display different behaviors in different settings. Different informants can help determine different disorder subgroups as well as variations in severity, prognosis, and etiology [24,26]. In addition, effective treatment planning and progress tracking depends on understanding how a child’s behavior differs in various contexts [27]. These implications have been well-documented for some disorders but differences in the area of SM are not as well known. 

Parents and teachers tend to rate children with SM differently [28]. Investigators indicated that while children with SM are often incorrectly referred to as shy, their most significant problems have involved social communication and educational achievement. For children with SM, social anxiety was identified in 74% of cases and generalized anxiety disorder was found in 11%. Results from the Conners’ Rating Scale found a significant mean difference in scores between parent and teacher reports of social problems, cognitive problems/inattention, and inattentiveness. For children with SM, teachers rated them as having significantly more social impairments than did their parents.

What causes these discrepancies is worthy of consideration. One factor is simply that children with SM behave differently in different contexts. Different portrayals of a child can be explained by informants observing behavior that varies with the setting, interactions, and the measures that were used. Investigators used observers to code disruptive behaviors between parents and children and then between examiners and the same children [24]. As one might assume, their data showed that 29% of preschoolers displayed disruptive behavior in the presence of parents alone, 15% with the examiner alone, and 9% for both parent and examiner interactions. Behaviors change in the presence of different people. Parents and teachers of children with symptoms of disorders such as autism complete multiple measures describing the child’s adaptive and clinical behaviors differently [29]. Investigators may find that behavioral questionnaires completed by parents and teachers over-identify autism in children with SM. Reduced social interactions and functional communication, common in SM, are also symptoms of autism. 

A meta-analysis by Stratis and Lecavalier [22] found greater interrater agreement when rating externalizing behaviors than internalizing behaviors. SM is more of an internalizing disorder. Murray, Ruble, Willis, and Molloy [27] conducted a study in which parents and teachers of 45 children and adolescents (ages 5–14 years) with ASD rated children on various social skills. The authors found that parents regularly rated children more positively when they initiated interactions, while teachers consistently rated children more positively when they maintained interactions. Parents and teachers may expect different behaviors from children and have different sets of experiences [24,27,29] affecting choices on rating scales.

One study with multiple informants for children with SM [30] explored differences of externalizing behavior versus internalizing behaviors in different situations and with different informants. Both parents and teachers rated children with SM as having significantly more internalizing behaviors. Children were thought to be more withdrawn, more anxious/depressed, have more social problems, more thought problems, and more attention problems. While it was clear that children with SM behaved differently from controls. Parents and teachers rated children’s internalizing behaviors significantly more problematic than typically-developing children matched for age, socioeconomic status, and geographical location.

### 1.6. Aims of This Study

The purpose of this study is to compare parents’ and teachers’ perceptions on clinical and adaptive rating scales of the Behavior Assessment System for Children (BASC-3) for children with a diagnosis of SM to: (1) determine if significant differences exist between parents’ and teachers’ ratings on composite scales of internalizing behaviors, externalizing behaviors, adaptive skills, and behavioral symptoms; (2) determine if significant relationships exist between parents’ and teachers’ ratings of children on scales of withdrawal, anxiety, social skills, and functional communication and children’s speaking frequency in different settings; and (3) determine if significant relationships exist between parents’ and teachers’ ratings of children on adaptive skills and standardized test scores of language competency.

We hypothesize that: (1) parents and teachers will rate children with SM differently on the BASC-3 scales with parents rating children as more impaired than teachers, (2) children with greater frequency of speaking in different settings will have less withdrawal and anxiety and better social skills and functional communication, and (3) teachers ratings of functional communication and social skills will positively correlate with standardized test scores of language development. 

## 2. Materials and Methods

### 2.1. Participants

Participants were recruited from a university community clinic serving individuals with communication disorders. The clinic was on campus at a private, mid-size metropolitan university in Pennsylvania. The families, referred by area hospitals, clinics, private practitioners, and regional schools, were seen for communication and behavioral evaluations related to SM. Of the 45 children evaluated, three did not meet the criteria for selective mutism. Diagnosis was determined by a licensed psychologist and speech-language pathologist team with more than 25 years’ experience and specialization in SM. Diagnosis was comprised of scores on the Behavior Assessment System for Children (BASC-3) with Structured Developmental History (SDH) [31], the Anxiety Disorders Interview Schedule (ADIS)-IV [32], Selective Mutism Questionnaire (SMQ) [33] and structured developmental interviews with parents. Evaluators verified that the DSM-5 criteria for SM were met. Exclusion criteria for the study included full-time special education services with a prior diagnosis of intellectual disability ASD or failing hearing or vision screening.

In total, 22 male and 20 female children from counties in Pennsylvania and New Jersey participated in the study. The mean age of the children was 7.07 years with a standard deviation (SD) of 2.4 years. Ages ranged from 2.4 years to 13.8 years. Fifty percent (50%) of the participants were in either prekindergarten or kindergarten. The oldest child was in 7th grade. All parents signed informed consent before the evaluation which was approved by the Institutional Review Board at the University. Sixty-four percent (64%) of participants were Caucasian, 14% were Asian, 14% were Hispanic, 3% were Eastern European, and 5% were listed as Other. For 83% of the children, English was their primary language. Other languages included Spanish (7%), Chinese (5%), Russian (2%) and Other (3%). Eighty-eight percent (88%) of families included 2-parent households. Concerning perceived severity of SM as rated by parents on the ADIS (Anxiety Disorders Interview Schedule)-IV [32], the mean level of interference that SM had on children’s lives, based on a scale from 0 to 8 with 8 being the most severe, was 6.4 (SD = 1.8). This indicated a moderately severe level of impact of SM on their lives.

### 2.2. Measures

Children in this study were assessed with a battery of speech-language measures and behavioral assessments [34] for a complete description of the study protocols).

The Peabody Picture Vocabulary Test (PPVT-4) [35], is a norm-referenced, standardized test to assess auditory comprehension for receptive vocabulary in single words. It is for individuals from 2 years and 6 months to 90+ years of age and covers 20 categories. Children point to respond when a word is said by the examiner. Internal consistency reliability was high (*r* = 0.93). The PPVT-4 is considered to be a valid receptive vocabulary measure.

The Expressive Vocabulary Test (EVT-2) [36], assesses expressive vocabulary. Participants name a picture. It is a norm-referenced, standardized test for individuals from 2 years and 6 months to 90+ years of age. It covers 20 categories of content and parts of speech. Internal consistency reliability was high (*r* = 0.93–0.94) as was test–retest reliability (*r* = 0.95). The EVT-2 is considered a valid expressive vocabulary measure.

The Test of Narrative Language [37,38] has two subtests, narrative comprehension (TNLc) and narrative production (TNLp). The TNL-2nd edition is for children from 5:0 through 15:11 years of age. It is a norm-referenced, standardized test measuring children’s ability to listen to stories, answer questions about stories, and tell stories. Tasks include three story types using a script, a personal narrative and a fictional narrative. Using the TNL-2, both receptive and expressive language competence can be tested. Internal consistency was good (*r* = 0.76–0.88) as was test–retest reliability (*r* = 0.85) and interrater reliability for scoring story transcripts (91%–98%).

The Test of Auditory Processing Skills serial number memory subtests (TAPS-3) [39] is a standardized and norm-referenced measure that assesses auditory serial memory in both forward and reversed number sequences. Children from 4 to 18 years listen to a series of numbers and repeat the sequences in order from recall. Items are ordered from easiest (less to recall) to more difficult. The TAPS-3 has good internal consistency with test–retest reliability of 0.96. It is considered a valid instrument for evaluating auditory processing skills.

Prior to arrival at the clinic, parents completed, and brought with them, the Behavioral Assessment System for Children–Structured Developmental History form and BASC-3 Parent Rating Scales and Teacher Rating Scales [31] as available. The BASC-3 is a multidimensional system assessing the behavior of children and young adults from 2 to 25 years of age. It includes a comprehensive set of rating scales that adds to a complete overview of children’s behavior. The Structured Development History provides a comprehensive history with developmental, educational, social, psychological, and medical background information. The Parent Rating Scale measures adaptive and problem behaviors in both home and community settings. Parents respond to 139–179 items based on age of the child. Responses are on a 4-point Likert scale indicating how frequently the child engages in a variety of behaviors from “never” to “almost always”. The Teacher Rating Scale measures adaptive and problem behaviors in the preschool or school environment. Between 105 and 165 items are answered on a 4-point scale ranging from “never” to “almost always”. Internal consistency was very good with coefficients from 0.82 to 0.97. Test–retest reliability was also good ranging from 0.80 to 0.93. One validity measure included an *F*-Index for which 96% to 99% of cases had acceptable interpretive ranges across rating scales. Validity included item analysis of 0.97 for parent and teacher rating scales [31].

The BASC-3 composite scales, rated by both parents and teachers, include four areas: (1) Internalizing Problems, (2) Externalizing Problems, (3) Adaptive Skills, and (4) Behavioral Symptoms Index. 

(1) Internalizing Problems include ratings for anxiety, depression, and somatization. Children with internalizing problems tend to be compliant and while not disruptive, peer relationships can suffer [31].

(2) Externalizing Problems include ratings for hyperactivity, aggression, and conduct problems. Externalizing problems involve disruptive behaviors. Children often come to the attention of school personnel which may go unresolved even with adult direction. 

(3) Adaptive Skills include ratings for adaptability, social skills, functional communication, and leadership. Adaptive skills summarize children’s ability to control themselves and express themselves emotionally in prosocial ways. Adaptive behaviors are important for home, school, and in public places for functional relationships. 

(4) The Behavioral Symptoms Index includes ratings for hyperactivity, aggression, depression, attention problems, atypicality, and withdrawal. The behavioral symptoms index relates to overall problem behaviors identified in interactional patterns and affect.

All scales are measured using *t*-scores with a mean of 50 and standard deviation of 10. Scores ranging from 40 to 60 fall within the average range. With the exception of the Adaptive Skills scale, scores between 60 and 69 are considered to be at-risk and scores above 70 are considered to be clinically significant. For the Adaptive Skills scale, scores from 31 through 40 are considered to be at-risk and scores 30 or lower are considered to be clinically significant. 

The Selective Mutism Questionnaire [33] is a 17-item parent questionnaire that measures frequency of talking in situations at school, home, and socially outside of school. It is used to determine the likelihood of a child having SM. A 4-point Likert scale offers options from 0 (never), 1 (seldom), 2 (often), to 3 (always). Estimates of internal consistency indicate good reliability ranging from 0.65 to 0.91 with total scale reliability coefficient (*r* = 0.78) for strong internal consistency. Convergent validity was supported with a clinician severity rating index from semi-structured clinical interviews [40].

The ADIS-IV was developed in connection with the DSM-IV: Child/Parent Versions [32] and provided a semi-structured interview format to help determine if a child meets the DSM criteria for SM and to what degree there is interference in the child’s life (rated from 0 to 8) where 4 indicates clinical significance. Overall ADIS has good test–retest reliability ranging from *r* = 0.61 to 1.00.

### 2.3. Procedures and Statistical Analysis

Parents contacted the University Clinic for Communication Sciences and Disorders requesting a communication evaluation for their children suspected of having SM or already having received a diagnosis of SM from a physician or clinical psychologist. Families learned about our clinic from a variety of sources including treatment professionals, school staff, clinical workshops, or word-of-mouth. After setting up the appointment with the clinic director, forms were sent home to families for completion to bring with them at the time of their evaluation appointment. Parents completed the BASC-3 Structured Developmental History, the BASC-3 Parent Rating Scale, and the SMQ. Parents gave the BASC -3 Teacher Rating Scale to their children’s teachers (during the school year) and asked them to complete the forms for return to the clinic. 

All children were accompanied to the evaluation by their parents or a parent and another familiar adult. When parents arrived at the clinic with their children, informed consent forms and release of information forms were reviewed and signed. One adult remained with the child while the professional prepped and trained the identified parent (or caregiver) how to read instructions and turn pages of the test booklets for assessment. Parents were alone in the testing room with their children while they delivered the test stimuli for vocabulary, narrative language, and auditory serial memory measures. Parents followed directions which only required the ability to read and flip pages of a booklet. They did not score any items. Professionals watched real-time from an observation room that was a few rooms away from the testing room. Video monitoring was consistent for accuracy with two professionals scoring real-time from the observation room. Video reviews also assisted if responses to any items were in question. Parents had directions beside them at all times for recall of what to say and do. All scoring and interpretations were completed by the licensed and certified professionals in the fields of speech-language pathology and psychology. Short breaks were given between each assessment measure. These included the PPVT-4 (receptive vocabulary), EVT-2 (expressive vocabulary), TNL-2 (narrative language comprehension and production), and TAPS-3 subtests (auditory serial memory recall forward and reversed).

After testing was complete, the parent(s) was asked to join the professionals for a debriefing session to discuss impressions and to ask questions. Children stayed with one of the adults that came to the evaluation while the other met with the professionals. Clinical graduate students were also available to play games if both parents wanted to meet with the professionals.

A follow-up consult was planned after the comprehensive evaluation report was completed. For a complete description of the validated testing process and outcomes from a prior study, see Klein, Armstrong, and Shipon-Blum [20]. 

The Statistical Package for the Social Sciences (SPSS 24) (IBM, Armonk, NY, USA) was used for all statistical analyses. 

## 3. Results

Results reflect the following three questions.

### 3.1. Are there Significant Differences between Parents and Teachers of Children with SM on Composite Scales of Internalization, Externalization, Adaptive Skills, and Behavioral Symptoms of the Behavior Assessment System for Children (BASC-3)? 

A 2x4 repeated measures factorial ANOVA examined the effects of rater (Parent and Teacher) on BASC-3 composite scales (Internalizing, Externalizing, Adaptive Skills, and Behavioral Symptoms) in Table 1. Greenhouse–Geisser corrections for degrees of freedom were reported for rater, *X^2^* (5) = 19.61, *p* = 0.001 and composite scale, *X^2^* (5) = 21.40, *p* = 0.001.

Main effects for Rater showed that parents rated their children with SM as having significantly more problems than teachers rated them, *F* (25, 1) = 5.34, *p* = 0.029. There was a large effect size of η^2^*p =* 0.176 for Rater. Although parent ratings indicated more problems on each of the four composite scales compared to teachers, paired *t*-test results revealed statistically significant differences on the Behavioral Symptoms Index only *t* (25) = 4.12, *p* < 0.000, which includes scales for withdrawal, atypicality, attention problems, depression, aggression, and hyperactivity. 

Main effects analysis for BASC-3 Composite Scales showed that children with SM displayed significantly more internalizing problems (anxiety, depression, and somatization) than externalizing problems (hyperactivity, aggression, and conduct problems). Collectively, the children also had greater difficulties on Adaptive Skills (social skills, functional communication, adaptability, leadership, study skills, and activities of daily living) than Behavioral Symptoms (hyperactivity, aggression, depression, attention problems, atypicality, and withdrawal). Statistically significant differences were identified, *F* (52.21, 2.09) = 16.15, *p* < 0.000. There was a large effect size of η^2^*p =* 0.392 for composite scales. No Interaction was found for Rater by Composite Scale, *F* (56.49, 2.26) = 2.89, *p* = 0.058.

### 3.2. What Is the Relationship between Parent and Teacher Ratings on Withdrawal, Anxiety, Social Skills, and Functional Communication of the BASC-3 and Frequency of Speaking in Various Settings Based on the Selective Mutism Questionnaire (SMQ)?

BASC-3 scores from parents of children with SM (*n* = 38) indicated clinically significant ratings for Withdrawal (*M* = 76.24, *SD* = 14.15) and at-risk ratings for Social Skills (*M* = 39.24, *SD* = 8.77), and at-risk ratings for Functional Communication (*M* = 39.11, *SD* = 10.35). Anxiety ratings fell within the average range (*M* = 57.32, *SD* = 15.28). BASC-3 ratings are based on T scores with a mean of 50 and SD of 10. BASC-3 scores from teachers of children with SM (*n* = 28) indicated at-risk ratings for Withdrawal (*M* = 65.64, *SD* = 11.08); at-risk ratings for Social Skills (*M* = 38.32, *SD* = 7.61), and at-risk ratings for Functional Communication (*M* = 39.96, *SD* = 12.16). Anxiety ratings fell within the average range (*M* = 55.93, *SD* = 10.89). Elevated scores on the Anxiety scale of the BASC-3 are not sufficient to diagnose an anxiety disorder [31]. Social Skills, and Functional Communication scales were areas rated as at-risk and Withdrawal was rated as clinically significant. Anxiety ratings were also included as SM is considered an anxiety-based disorder according to the DSM-5. Table 2 presents correlations for parent and teacher ratings with frequency of speaking based upon scores on the SMQ for school, home, and social settings. SMQ frequency of speaking ranges from 0 to 3 with higher numbers related to greater frequency of speaking, more like typical children.

The more withdrawn children were in school, the less they spoke. Less speaking in school was significantly correlated with greater withdrawal ratings by teachers, *τ* (22) = −0.46, *p* = 0.004 and parents *τ* (29) = −0.32, *p* = 0.020. Children with higher scores on social skills, rated by teachers, spoke more frequently in school *τ* (22) = 0.52, *p* = 0.001. According to parents, children with higher withdrawal ratings spoke less in social settings outside of school, *τ* (29) = −0.46, *p* = 0.001.

Paired *t*-test analyses were conducted to determine differences between parents’ and teachers’ ratings on children’s Withdrawal, Anxiety, Social Skills, and Functional Communication. Table 3 provides means, standard deviations, levels of significance, and Cohen’s *d* effect sizes.

Parents and teachers rated the same children as significantly different on the Withdrawal scale *t* (26) = 3.26, *p* = 0.003. Parents viewed their children as significantly more withdrawn than did their teachers. Parents and teachers rated children with SM similarly on scales measuring anxiety, social skills and functional communication. 

### 3.3. What Is the Relationship between Parent and Teacher Ratings on Adaptive Skills of the BASC-3 (Adaptability, Social Skills, Functional Communication, Leadership, Study Skills, and Activities of Daily Living) and Standardized Measures of Receptive Vocabulary (PPVT-4), Expressive Vocabulary (EVT-2), Comprehension and Production of Narrative Language for Stories (TNL-2), and Auditory Serial Memory (TAPS-3 Numbers Forward and Reversed)? 

Kendall’s *tau-b* correlations in Table 4 show a statistically significant relationship between teacher ratings on the Adaptive Skills Composite Scale for five of six language measures (receptive and expressive vocabulary, comprehension of narrative language, and auditory serial memory forward and reversed). Language measures were standardized and norm-referenced. Parents did not associate adaptive skills with any language skills.

Children with better adaptive skills were associated with: higher vocabulary comprehension, τ (26) = 0.34, *p* = 0.019; higher expressive vocabulary naming, τ (25) = 0.42, p = 0.004; higher comprehension of narrative language for stories, τ (22) = 0.51, p = 0.002; higher auditory serial memory for numbers forward, τ (23) = 0.63, *p* < 0.000; and higher auditory serial memory for numbers reversed, τ (22) = 0.44, *p* = 0.006 according to teacher ratings. 

Kendall’s *tau-b* correlations were calculated for tests of language with teachers’ and parents’ ratings for withdrawal, anxiety, social skills, and functional communication. Table 5 shows that teachers rated children who were less withdrawn as having better vocabulary comprehension, *τ* (27) = 0.34, *p* = 0.015. Children with better social skills were also found to have higher vocabulary scores on the PPVT-4, *τ* (27) = 0.28, *p* = 0.044. Better social skills were also associated with higher scores on narrative language comprehension on the TNL-2, *τ* (23) = 0.33, p = 0.038. Greater serial auditory memory for numbers forward on the TAPS-3 was associated the less withdrawal, *τ* (24) = −0.37, *p* = 0.016, and greater social skills, *τ* (24) = 0.38, *p* = 0.014, as rated by teachers. Parents’ ratings on the same BASC-3 scales were not significantly correlated with any measures of vocabulary, narrative language, or serial auditory memory. 

We were correct in our hypotheses that: (1) parents and teachers rated children with SM differently on the BASC-3 composite scales with parents rating children as more impaired than teachers, (2) children with greater frequency of speaking in different settings had less withdrawal and anxiety and better social skills and functional communication, and (3) teachers identified relationships between language skills and adaptive skills more successfully than parents. 

## 4. Discussion

Overall, parents and teachers rated children with SM differently on a number of measures. Parents rated their children with SM as having more problems on the Composite Scales of the BASC-3 (internalization, externalization, adaptive skills, and behavioral symptoms) than did teachers. Parents also rated their children as having significantly more behavioral symptoms than teachers. As parents, they have the opportunity to observe their children in a greater range of environments and social settings than teachers. Teachers, on the other hand, see children only in school where the day is largely controlled. School is more challenging because children are away from the comfort of what is considered safe and familiar at home [41]. There is also an element of performance anxiety in school with classes containing larger groups of people, many of whom are initially unfamiliar. Demands for communication also vary from home to school [42]. As part of a larger class, children with SM often remain quiet. Conversely, parents see their children in more varied settings that are less structured. Children who remain quiet during the long school hours come home frustrated after not speaking all day. Anxiety, depression, and somatization can result [43]. It is not uncommon for children with SM to have more internalizing behaviors at school and externalizing behaviors at home. During the assessment process for this study, parents noted that their children became more demanding at home after the day at school.

In this study, children with SM displayed significantly more internalizing problems (anxiety, depression, and somatization) than externalizing ones (hyperactivity, aggression, and conduct problems) and significantly more problems with adaptive skills (functional communication, social skills, adaptability, leadership, and study skills) than Behavioral Symptoms (hyperactivity, aggression, depression, attention problems, atypicality, and withdrawal). Behavioral Symptoms presented the significantly different perceptions between parents and teachers with parents conveying more problems in children with SM. The most prominent behavioral symptom was withdrawal.

Communication was a major deficit area for this population [20]. Children tended to be followers and rarely initiated interactions, especially if the interactions required talking. Several children wrote that they wanted to talk but could not. Combined with lower scores on Social Skills and Functional Communication scales, 80% of the children in this study received a diagnostic label of ASD (299.00, F84.0) on the BASC-3 Interpretive Summary Report [31]. Items such as: *Is shy with other children, Avoids other children, Has trouble making new friends, and Is shy with adults* were commonly identified by parents and teachers. Anxiety was also a common concern found in children with ASD [44].

The Autism Probability Index of the BASC-3 is comprised of items from scales of Withdrawal, Atypicality, Functional Communication, Leadership, and Social Skills. Children with elevated scores on the Index displayed behaviors that were considered unusual, such as not talking when outside their comfort zone. Children with SM often received elevated scores related to problems developing and maintaining social relationships [45]. Although children with SM spoke more frequently at home with family, they were rated as withdrawn in most other settings and fell within the clinically significant range by parents and at-risk range by teachers [33].

According to Steffenbeurg, Steffenburg, Gillberh, and Bellstedt [4], children with SM, who were also diagnosed with ASD, tended to be older at the age of diagnosis, had a history of speech delay, had a later onset of symptoms, and a greater proportion also had intellectual disability.

The more children spoke at home, the higher parents rated their functional communication in general. For parents of children who did not speak in public, their children were rated as more withdrawn. Parents conveyed despair for their children who did not speak once they left the comfort of their home or car. Parents rated their children as more withdrawn than teachers. This may, in part, be due to parents having the benefit of observing their children in different settings, seeing the contrast. Teachers only observed them in the school environment where quiet children do not call attention to themselves. Another explanation is that children interact more nonverbally with peers in school and are therefore viewed as less withdrawn, even if they do not speak. 

Teachers identified some children with SM as having learning problems. For those children, their ability to pay attention was a major concern. One would think that sitting quietly means you are paying attention. This did not seem to be the case. Children with SM are often in a state of hypervigilance much of the school day, monitoring their surroundings for fear of being called on, worrying about being asked questions, or feeling trepidation about others’ attempts to try to get them to talk [46]. Such energy can take time away from focused attention for class instruction. Children with SM who were rated as speaking more frequently in school on the SMQ were rated as having better social skills on the BASC-3 by teachers [31].

Teachers’ ratings on BASC-3 adaptive skills were significantly and positively correlated with standardized, norm-referenced test scores of language. Communication deficits have been identified in children with more severe levels of SM [20,30,47]. For children who do not get adequate practice talking to various people in a variety of settings, skills can become deficient. Children need practice. Children who scored lower on language measures were rated as having more behavioral symptoms with poorer attention, greater withdrawal, atypicality, hyperactivity, and aggression.

Parents’ ratings on adaptive skills were not significantly correlated with any of the language measures. Parents often focused on whether or not their children were talking. Content of speech was not the primary concern for most parents. More advanced language skills such as using complex sentence structures, engaging in decontextualized language, initiating conversations, answering Wh-questions (who, what, where, when, why, and how), and speaking with a sufficient mean length of utterance did not appear to be on parents’ radar. Rather, “talking” became the main focus for parents. Children’s deficits became more obvious in the school setting where teachers had a peer comparison group. Both parents’ and teachers’ input is necessary when analyzing the complexity of SM, as parents and teachers see children with SM through different lenses.

The empirical results within this study should be considered in relation to some limitations. All participants were part of a clinical population. Children and their parents came to a Communication Sciences and Disorders University Clinic that specializes in SM. Parents brought their children for testing due to their concerns about mutism affecting their children’s lives in and out of school. The sample population tended to be a more moderate to severe group of children with SM. This lead to a possible lack of probability sampling. However, with a disability group receiving evaluations, it is not uncommon. Some children were home-schooled which prevented obtaining teacher rating scales. Other children came to the clinic during the summer months which prevented obtaining those teachers’ rating scales. There were 15 teacher ratings and 15 parent ratings used for within-subject analyses. Rating scales were matched. Nonparametric analyses were used. All children and their families came from two Mid-Atlantic States, Pennsylvania and New Jersey. This may limit generalizability to all regions of the United States. Future investigators may decide to include more children and consider using more than one behavioral rating scale. Clinical researchers may also want to administer a diagnostic measure such as the Autism Diagnostic Observation Scale (ADOS-2) to determine actual concordance rates of children identified as having both SM and ASD. Updated measures are needed to accurately screen for ASD with greater precision [45] in children with SM.

## 5. Conclusions 

SM is more than shyness. It is considered an anxiety-based disorder that affects communication. Children with SM usually want to talk but cannot do so in situations where they feel an expectation to talk and where the people and places are less than familiar. Proper diagnosis is crucial for effective treatment. Based on the results of this study, we found both parents and teachers to be essential contributors on behavioral rating scales when diagnosing children with SM. Parents and teachers provided different views of behavioral symptoms at school, home, and in social settings outside of school. Withdrawal was identified as being the most prominent feature, with parents rating children as more impaired than teachers rated them. Both groups identified social skills and functional communication to be clinically at-risk. Teachers accurately identified areas of vocabulary, narrative comprehension for stories, and auditory serial memory as concerns in children with SM based on standardized test scores. Parents were found to be less aware of academic language expectations and the relationship of those skills to adaptive skills.

Children with SM were often identified as having ASD on the BASC-3 interpretive summary report. While it is possible to have a concomitant disorder of ASD with SM, developers of questionnaires are encouraged to include items that can identify and differentiate variations in social communication in different settings with various people. White, Bray, and Ollendick [45] noted that the two conditions can co-occur but that it is crucial to measure factors to identify restricted interests and routine preferences, common to ASD.

Of great importance is the fact that the longer children with SM remain without treatment, the more difficult gains from treatment can become [48]. Practitioners and researchers can help increase awareness to educate parents and teachers about this complex childhood disorder. We need input and collaboration from all involved to achieve a holistic understanding of the child, based on the different perspectives each one provides.

## Figures and Tables

**Table 1:** Paired *t*-test for Behavior Assessment System for Children (BASC) Composite Scales with Raters for 26 children with Selective Mutism (SM). SD; Standard Deviation.

Measures Mean = 50 (SD = 10)	Parents Mean (SD)	Teachers Mean (SD)	*t*	*p*	*d Effect Size*
Internalizing	53.00 (11.21)	50.27 (7.69)	*t* (25) = 1.16	0.258	0.28 small
Externalizing	45.15 (9.54)	42.54 (4.41)	*t* (25) = 1.36	0.186	0.35 small
Adaptive Skills	41.77 (8.58)	42.46 (8.42)	*t* (25) = −0.348	0.731	0.08 none
Behavioral Symptoms	54.58 (8.14)	47.77 (4.96)	*t* (25) = 4.12	0.000 **	1.01 large

*p* < 0.05. ** *p <* 0.01.

**Table 2:** Kendall’s *tau-b* correlations for BASC-3 scales (Mean = 50, SD = 10) and Selective Mutism Questionnaire (SMQ) frequency of speaking at school, home, and in social settings outside of school.

Questionnaire Scales	Mean (SD) *n*	1	2	3	4	5	6	7	8	9	10	11
1. SMQ School Range 0–3	0.82/3.0 (0.68) 31											
2. SMQ Home Range 0–3	2.0/3.0 (0.59) 31	−0.01										
3. SMQ Social Range 0–3	0.51/3.0 (0.54) 31	0.49 **	−0.40 **									
4. Withdr (Parent) *M* = 50 (*SD* = 10)	*T* = 76.24 (14.15) 38	−0.32 *	−0.15	−0.46 **								
5. Withdr (Teacher) *M* = 50 *(SD =* 10)	*T* = 65.64 (11.08) 28	−0.46 **	0.26	−0.28	0.34 *							
6. Social Skills (P) *M* = 50 *(SD =* 10)	*T* = 39.24 (8.77) 38	−0.19	0.20	0.09	−0.17	0.07						
7. Social Skills (T) *M* = 50 *(SD =* 10)	*T* = 38.32 (7.61) 28	0.52 **	−0.10	0.23	0.01	−0.22	0.05					
8. Funct Comm (P) *M* = 50 *(SD =* 10)	*T* = 39.11 (10.35) 38	−0.07	0.23	0.10	−0.09	0.04	0.50 **	0.13				
9. Func Comm (T) *M* = 50 *(SD =* 10)	*T* = 36.96 (12.16) 28	0.23	−0.01	0.12	0.08	−0.24	0.09	0.45 **	0.10			
10. Anx (P) *M* = 50 *(SD* = 10)	*T* = 57.32 (15.28) 38	−0.01	0.03	−0.05	0.25 *	0.27	0.11	−0.04	−0.09	0.03		
11. Anx (T) *M* = 50 *(SD =* 10)	*T* = 55.93 (10.89) 28	−0.24	0.14	−0.13	0.24	0.51 **	0.13	−0.15	0.06	−0.12	0.28 *	

* *p* < 0.05; ** *p* < 0.01.

**Table 3:** Paired *t*-Test results with Means and SDs for 26 pairs of parents and teachers on BASC-3 scales.

Measure Mean (SD)	Parent Rating Mean (SD)	Teacher Rating Mean (SD)	*t*	*p*	*d* Effect Size
Withdrawal *M* = 50 (10)	74.81 (14.89)	65.63 (11.29)	t (26) = 3.26	0.003 **	0.69 medium
Anxiety *M* = 50 (10)	55.48 (12.58)	56.07 (11.07)	t (26) = 2.29	0.821	0.05 none
Social Skills *M* = 50 (10)	38.41 (7.99)	37.85 (7.33)	t (26) = 2.75	0.786	0.07 none
Functional Communication *M* = 50 (10)	40.52 (10.86)	36.81 (12.36)	t (26) = 1.29	0.206	0.32 small

*p* < 0.05. ** *p* < 0.01.

**Table 4:** Kendall’s *tau-b* correlations for vocabulary (Peabody Picture Vocabulary Test (PPVT) and Expressive Vocabulary Test (EVT)), narrative language (Test of Narrative Language-comprehension (TNL-C) and Test of Narrative Language-production (TNL-P)), and auditory memory measures (Test of Auditory Processing Skills (TAPS)-F and TAPS-R) with parent (P) and teacher (T) ratings on the BASC-3 Adaptive Skills composite scale.

Measure Mean (SD)	Mean (SD) *n*	1	2	3	4	5	6	7	8
1.PPVT Rec *M* = 100 (15)	99.29 (16.91) 41								
2. EVT Exp *M* = 100 (15)	99.66 (17.24) 38	0.52 **							
3. TNL Comp *M* = 10 (3)	9.41 (3.69) 34	0.37 **	0.47 **						
4. TNL Prod *M* = 10 (3)	8.06 (3.85) 34	0.30 *	0.30 *	0.40 **					
5. TAPS For *M* = 10 (3)	9.54 (3.31) 35	0.35 **	0.35 **	0.45 **	0.22				
6. TAPS Rev *M* = 10 (3)	8.73 (4.13) 33	0.36 **	0.36 **	0.42 **	0.24	0.56 **			
7. Adapt (P) *M* = 50 (10)	40.76 (8.79) 37	0.10	0.17	−0.01	0.15	0.18	0.14		
8. Adapt (T) *M* = 50 (10)	42.56 (8.27) 27	0.34 *	0.42 **	0.51 **	0.20	0.63 **	0.44 **	0.23	

* *p* < 0.05. ** *p* < 0.01.

**Table 5:** Kendall’s *tau-b* Correlations of BASC-3 Adaptive Skills Composite Scale as Rated by Teachers (T) and Parents (P) and Standardized Language Measures.

Measure Mean (SD)	Mean (SD) *n*	1	2	3	4	5	6	7	8	9	10	11	12	13	14
*1. Withdr(T)* M *= 50 (10)*	65.64 (11.08) 28														
*2. Anxiety(T)* M *= 50 (10)*	55.93 (10.89) 28	0.51 **													
*3. SocSkill(T) * M *= 50 (10)*	38.32 (7.61) 28	−0.22	−0.15												
*4. FComm(T) * M *= 50 (10)*	36.96 (12.16) 28	−0.24	−0.12	−0.45 **											
*5. Withdr(P) * M *= 50 (10)*	76.24 (14.15) 38	0.34 *	0.24	0.01	0.08										
*6. Anxiety(P) * M *= 50 (10)*	57.32 (15.28) 38	0.27	0.28 *	−0.04	0.03	0.25 *									
*7. SocSkill(P) * M *= 50 (10)*	39.24 (8.77) 38	0.07	0.13	0.05	0.09	−0.17	0.11								
*8. FComm(P)* M *= 50 (10)*	39.11 (10.35) 38	0.04	0.06	0.13	0.10	−0.09	−0.09	0.50 **							
*9. PPVT* M *= 100 (15)*	99.29 (16.91) 41	−0.34 *	−0.08	0.28 *	0.10	−0.08	−0.17	−0.02	0.15						
*10. EVT* M *= 100 (15)*	99.66 (17.24) 41	−0.25	−0.20	0.21	0.13	−0.17	−0.15	0.08	0.19	0.52 **					
*11. TNLc* M *= 10 (3)*	9.41 (3.69) 34	−0.28	−0.27	0.33 *	0.13	0.09	−.08	−0.20	0.01	0.37 **	0.47 **				
*12. TNLp* M *= 10 (3)*	8.06 (3.85) 34	−.24	−0.05	0.12	0.05	−0.24	0.13	0.12	0.20	0.30 *	0.30 *	0.40 **			
*13. TAPSf* M *= 10 (3)*	9.54 (3.31) 35	−0.37 *	−20	0.38 *	0.17	0.04	−0.12	0.01	0.25	0.35 **	0.35 **	0.45 **	0.22		
*14. TAPSr* M *= 10 (3)*	8.73 (4.13) 33	−0.26	−0.03	0.24	0.13	0.09	−0.13	−0.07	0.14	0.36 **	0.36 **	0.42 **	0.24	0.56 **	

** Correlation is significant at the 0.01 level (2-tailed). * Correlation is significant at the 0.05 level (2-tailed). Number Codes: BASC-3 (T) = Teacher Ratings for: 1-Withdrawal, 2-Anxiety, 3-Social Skills, 4-Functional Communication; BASC-3 (P) = Parent Ratings for 5-Withdrawal, 6-Anxiety, 7-Social Skills, 8-Functional Communication; Language Measures for: 9- PPVT-4 (Receptive Vocabulary), 10-EVT-2 (Expressive Vocabulary), 11-TNL-2 (c = Comprehension of Narrative Language), 12-TNL-2 (p = Production of Narrative Language), 13-TAPS-3 (f = Auditory Number Memory Forward), 14- TAPS-3 (r = Auditory Number Memory Reversed).
